# MRGPRX2-mediated mast cell activation by substance P from overloaded human tenocytes induces inflammatory and degenerative responses in tendons

**DOI:** 10.1038/s41598-024-64222-1

**Published:** 2024-06-12

**Authors:** Rouhollah Mousavizadeh, Charlie M. Waugh, Robert G. McCormack, Brian E. Cairns, Alex Scott

**Affiliations:** 1 Department of Physical Therapy, Centre for Aging SMART, Centre for Aging SMART, University of British Columbia, 2635 Laurel Street, Vancouver, BC V5Z 1M9 Canada; 2 Department of Orthopaedics, University of British Columbia, Vancouver, BC Canada; 3 Faculty of Pharmaceutical Sciences, University of British Columbia, Vancouver, BC Canada

**Keywords:** Tendons, Chronic inflammation

## Abstract

Mast cells are immune cells minimally present in normal tendon tissue. The increased abundance of mast cells in tendinopathy biopsies and at the sites of tendon injury suggests an unexplored role of this cell population in overuse tendon injuries. Mast cells are particularly present in tendon biopsies from patients with more chronic symptom duration and a history of intensive mechanical loading. This study, therefore, examined the cross talk between mast cells and human tendon cells in either static or mechanically active conditions in order to explore the potential mechanistic roles of mast cells in overuse tendon injuries. A coculture of isolated human tenocytes and mast cells (HMC-1) combined with Flexcell Tension System for cyclic stretching of tenocytes was used. Additionally, human tenocytes were exposed to agonists and antagonists of substance P (SP) receptors. Mast cell degranulation was assessed by measuring β-hexosaminidase activity. Transwell and cell adhesion assays were used to evaluate mast cell migration and binding to tendon extracellular matrix components (collagen and fibronectin), respectively. Gene expressions were analyzed using real time qRT-PCR. Our results indicate that mechanical stimulation of human tenocytes leads to release of SP which, in turn, activates mast cells through the Mas-related G-protein-coupled receptor X2 (MRGPRX2). The degranulation and migration of mast cells in response to MRGPRX2 activation subsequently cause human tenocytes to increase their expression of inflammatory factors, matrix proteins and matrix metalloproteinase enzymes. These observations may be important in understanding the mechanisms by which tendons become tendinopathic in response to repetitive mechanical stimulation.

## Introduction

Repetitive-use tendinopathy is a persistent and painful condition, usually characterized by structural changes in tendon tissue indicative of repetitive cycles of injury and repair, such as accumulation of Type III collagen and neovascularization. While traditional inflammatory cells such as neutrophils and macrophages are minimally present, there is a notable abundance of mast cells (MCs) in human tendinopathic patellar tendon (PT) tissue^1–3^. However, the underlying mechanism for this occurrence remains unclear.

MCs are a component of the immune system found in connective tissues, including tendons. They originate from the migration and differentiation of myeloid progenitor stem cells induced by factors such as c-kit ligand and stem cell factor. The granules in mast cells store various factors, including inflammatory mediators and proteolytic enzymes. Activation of MCs leads to degranulation, subsequently releasing inflammatory factors^4^. These factors changes the expression of ECM remodelling proteins, fibrotic factors ad inflammatory mediators in tendon cells, thereby potentially contributing to tendon injury-repair mechanisms^5^. In chronically injured tendon tissue, we have observed that MCs accumulate in the hypervascular endotendon and paratendon cell layers^2^. In general, the loose connective tissue layers of tendon respond most actively following injury with cellular proliferation and inflammatory cell migration, cytokine and growth factor expression, and matrix remodeling^6,7^. In both acute injuries and chronic repetitive use tendinopathies, excessive proliferation of paratendon and endotendon contributes to a loss of normal tendon structure, with an expansion of neurovascular tissue and MCs. Thus, tendinopathic tendon is typically characterized as thickened (increased cross-sectional area) with inferior mechanical properties^8,9^.

Despite the well-established role of MCs in contributing to tissue repair in acutely injured soft tissues, their impact on tendon cell and tissue function within the mechanically dynamic environment of chronic tendon injuries has not been fully explored. MCs in connective tissues are exposed to a distinctive environment, experiencing repetitive and intense strains from muscle activation within constrained spaces where tendon thickening and edema can lead to further irritation and low-grade inflammation. Mechanical stress can directly induce mast cell degranulation^10^. However, it's important to note that tenocytes are the predominant resident cells in the tendon tissue and mast cells are scarce in healthy tendon tissue^2,11^. Therefore, tenocytes are potentially responsible for biological outcomes in response to mechanical stress.

Notably, tenocytes, being mechanically responsive cells, have the ability to sense and respond to applied strains^12,13^. This response promotes the release of factors that may activate neighboring cells, including MCs in the surrounding connective tissue. Previous studies have indicated that mechanical stimulation enhances Substance P (SP) expression by tenocytes^14^. The binding of SP to Mas-related G-protein-coupled receptors X2 (MRGPRX2) on MCs triggers cell activation and degranulation characterized by the release of cytoplasmic granule contents, including inflammatory mediators, into the extracellular space. This event leads to elevation of cytokines and the recruitment of immune cells, including CD45 + cells, neutrophils, and monocytes, ultimately promoting inflammation and contributing to painful conditions^15–17^. However, the potential for this mechanism to play a role in tendons has not been investigated. This study explored a possible mechanistic role of MRGPRX2 in the cross-talk between human tenocytes and HMC-1 (a cell line with MC characteristics)^18^, aiming to identify mechanisms that may contribute to the development of tendinopathy. We hypothesized that mechanical stress stimulates human tenocytes to release SP which activates MRGPRX2 on mast cells, ultimately leading to the recruitment and degranulation of mast cells—an event that potentially promotes inflammation and pain in tendinopathy.

## Results

### Repetitive stretching induces expression and release of factors by tenocytes, enhancing mast cell activity

**Figure 1:**
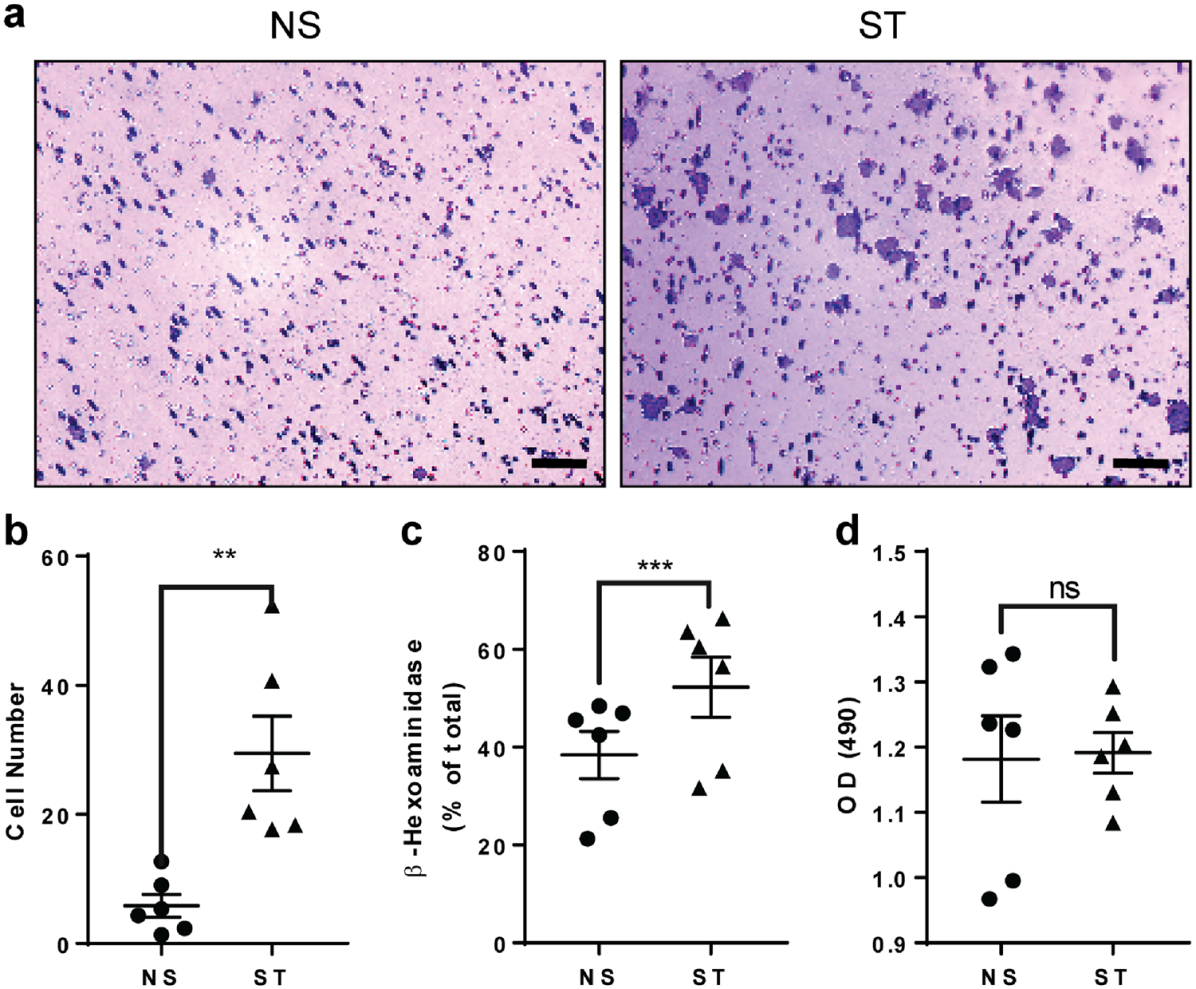
Tenocytes subjected to repetitive stretch recruit and degranulate MCs.Mechanical stretching (ST) of human tenocytes releases factors in conditioned media that increase HMC-1 migration as detected with crystal violet (**a**). Counting the number of cells which migrate across the transwell barrier showed that the changes are significant (**b**) compared to non-stretched (NS) cells. These factors also increased HMC-1 degranulation (**c**) with no changes in cell proliferation (**d**). Scale bars = 50 μμm.Paired t test; mean ± SE; **P ≤ 0.01; ***P ≤ 0.001; n = 6.

**Figure 2:**
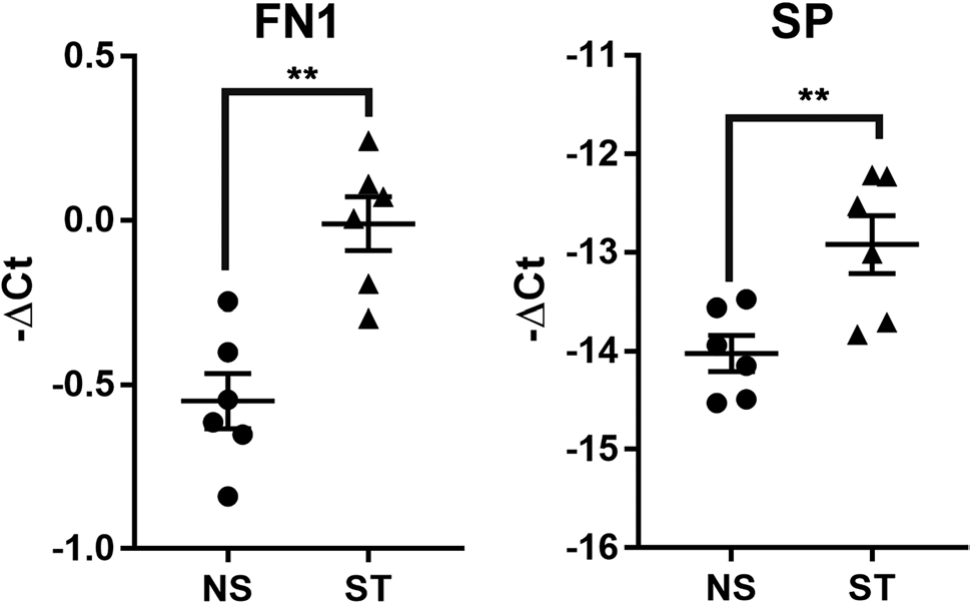
Increased expression of MC activators in stretched tenocytes. Mechanical stretching (ST) of human tenocytes significantly increases mRNA expression of MC activators, fibronectin 1 (FN) and Substance P (SP), compared to the non-stretched cells (NS). Paired t test; mean ± SE; **P ≤ 0.01; ***P ≤ 0.001; n = 6.

Tenocytes are mechanically responsive cells, capable of sensing and responding to applied strains. We used a Flex cell system to apply controlled, cyclic, tensile strains to adherent culture of human tenocytes. Upon repetitive cyclic stretching, human tenocytes released factors into the media which promoted HMC-1 migration and degranulation (Fig. 1). We next examined the gene expression of factors in stretched human tenocytes that could activate MCs. Previous data indicate that human tenocytes are capable of producing fibronectin and substance proteins^14,19^, which are factors known to activate mast cells^20,21^. The qPCR analysis revealed elevated expression of SP and fibronectin in stretched tenocytes at the mRNA level (Fig. 2), suggesting their potential involvement in mast cell migration and degranulation.

### Substance P receptor, MRGPRX2, promote mast cell degranulation and migration

**Figure 3:**
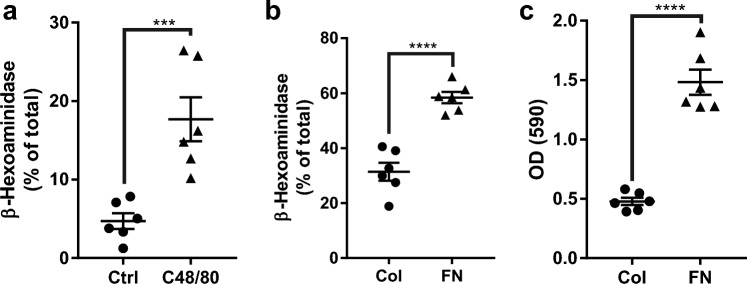
Influence of MRGPRX2 agonist and Fibronectin and on HMC-1 degranulation and adhesion. Adding agonist of MRGPRX2 (C48/80) to HMC-1 culture significantly increased degranulation (**a**). HMC-1 culture in fibronectin (FN) coated wells showed increased degranulation (**b**) and adhesion (**c**) compared to collagen (Col). Paired t test; mean ± SE; ***P ≤ 0.001; ****P ≤ 0.0001; n = 6.

**Figure 4:**
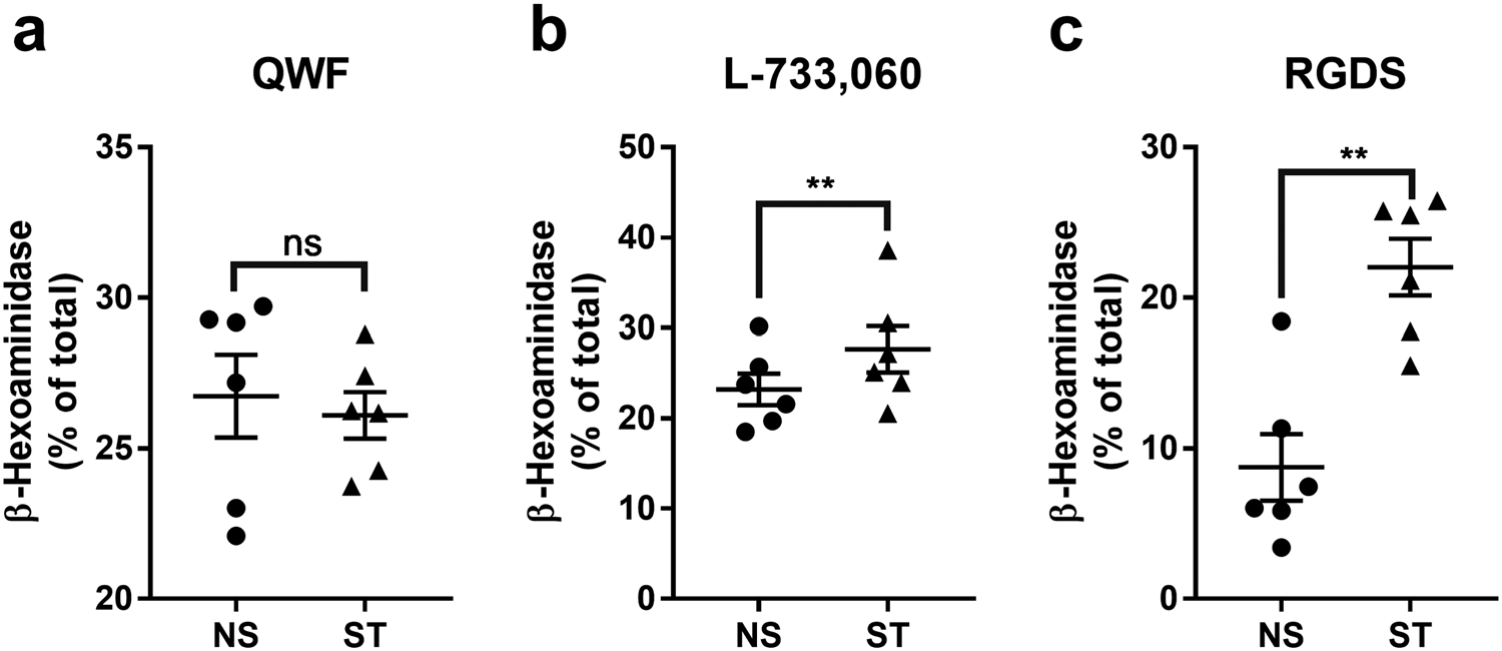
The influence of SP receptor antagonists on MC degranulation. MRGPRX2 antagonist (QWF) prevents MC degranulation induced by stretched tenocytes (**a**) while NKR1 antagonist (L-733,060) and RGDS failed to achieve the same effect (**b**, **c**). Paired t test; mean ± SE; **P ≤ 0.01; n = 6.

**Figure 5:**
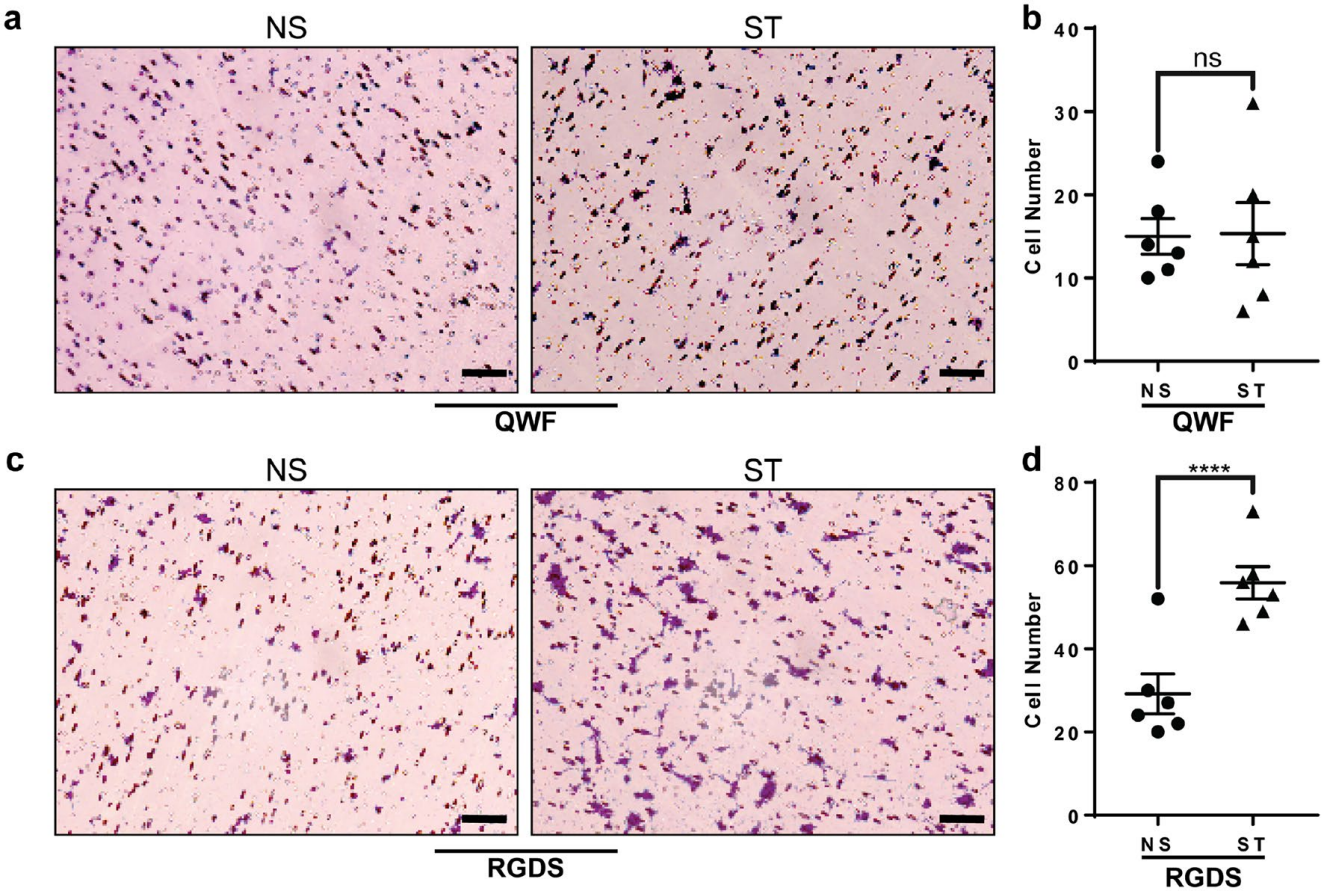
The effects of MRGPRX2 antagonist and RGDS on MC migration. The MRGPRX2 antagonist (QWF) inhibits MC migration induced by stretched (ST) human tenocytes compared to non-stretched (NS) tenocytes, as demonstrated in transwell micrographs (**a**, **b**). In contrast, RGDS failed to inhibit this process (**c**, **d**). Scale bars = 50 μμm. Paired t test; mean ± SE; ****P ≤ 0.0001; n = 6.

SP interacts with two distinct receptors on MCs, MRGPRX2, and NKR1^15^. HMC-1 cell line expresses functional MRGPRX2 receptor^22^. To specifically target MRGPRX2, we exposed HMC-1 to C48/80^23,24^, resulting in MC degranulation (Fig. 3 a). HMC-1 is a cell line derived from a mast cell leukemia patient and expresses MRGPRX2^22,25^. Fibronectin also induced HMC-1 degranulation and adhesion (Fig. 3b,c). We inhibited MRGPRX2 and NKR1 with the antagonists QWF^26^ and L-733,060^27^, respectively. QWF is a tripeptide SP antagonist with a low molecular weight and inhibits the binding of SP to MRGPRX2^28^. On the other hand, L-733,060 is a piperidine ether-based antagonist that targets the tachykinin NK1 receptor^27^. We also used RGDS to block the binding of MCs to fibronectin via integrins^20^. RGDS, a peptide sequence (arginyl-glycyl-α-aspartyl-serinyl) embedded in matrix proteins, interacts with integrins. These peptides have the ability to bind to integrins and prevent their interaction with extracellular matrix (ECM) proteins and ligands^29^. Our results showed that only QWF prevented the degranulation of HMC-1 cells after exposure to the conditioned media from stretched human tendon cells (Fig. 4). Blocking MRGPRX2 with QWF also abrogated HMC-1 migration in response to the conditioned media of stretched tenocytes (Fig. 5a,b), but RGDS did not affect this process (Fig. 5c,d).

### Activation of HMC-1 via MRGPRX2 promotes expression of inflammatory factors by human tenocytes

**Figure 6:**
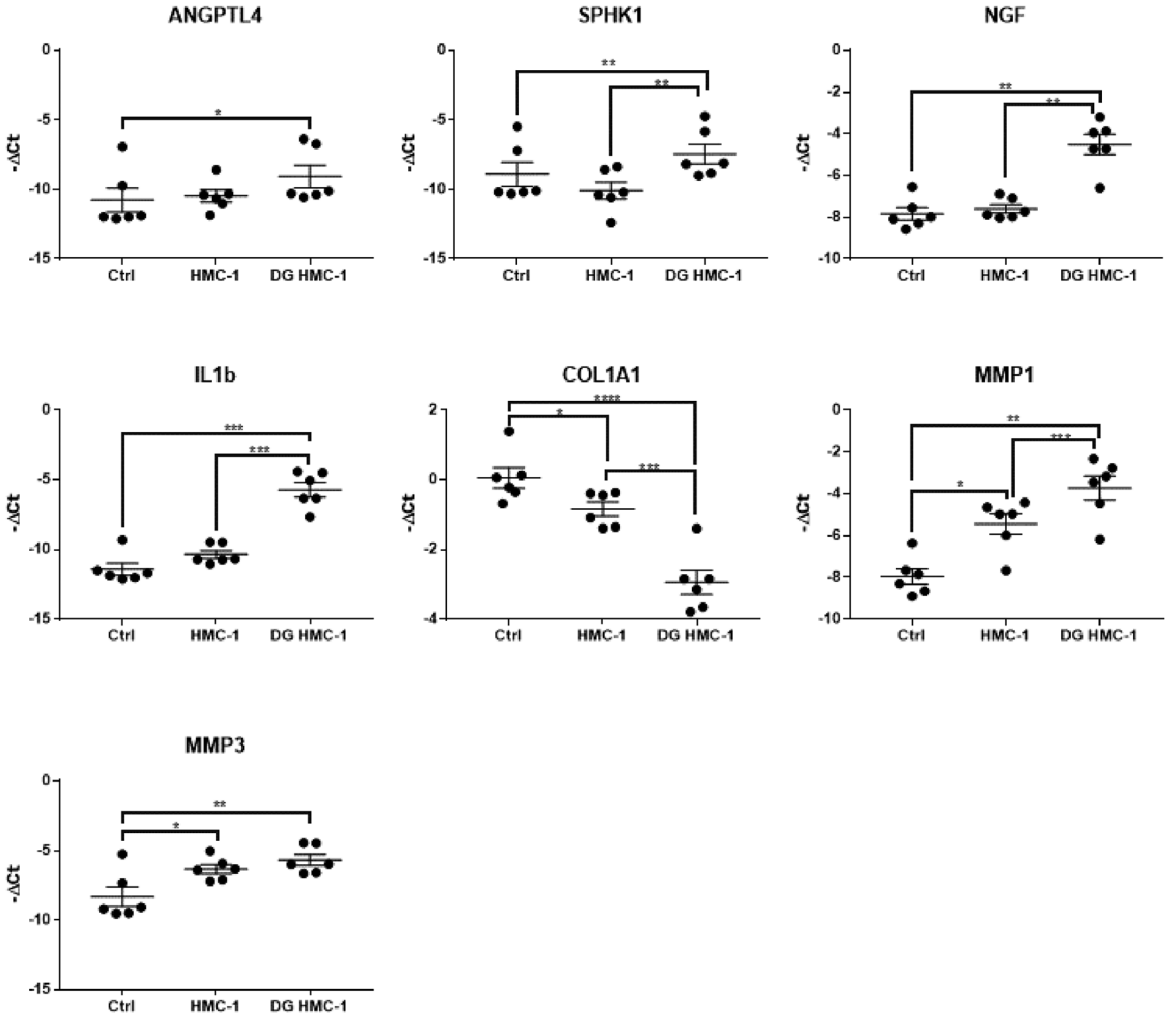
The influence of HMC-1 conditioned media on tenocyte gene expression upon activation by MRGPRX2 agonist. The conditioned media from HMC-1 induces changes in gene expression in human tenocytes compared to control (Ctrl). Particularly, upon degranulation of HMC-1 (DG HMC-1) by C48/80, the conditioned media exhibits a synergistic effect on gene expression in tenocytes. ANGPTL4: Angiopoietin-like 4; SPHK1: Sphingosine kinase 1; NGF: Nerve growth factor; Col1a1: Collagen type I αα1 chain; MMP1: Matrix metalloproteinase-1; MMP3: Matrix metalloproteinase-3; IL1b: Interleukin-1 β One-way ANOVA followed by a post hoc Tukey's test; mean ± SE; *P ≤ 0.05; **P ≤ 0.01; ***P ≤ 0.001; ****P ≤ 0.0001; n = 6.

HMC-1 were stimulated with a MRGPRX2 agonists (C48/80), and human tenocytes were then exposed to the conditioned media of the stimulated HMC-1 cells. The gene expression analysis of human tenocytes indicated changes in gene expression after exposure to the media containing HMC-1-released factors. Upon stimulation with MRGPRX2 agonists we observed a synergistic effect which shows significantly increased expression of angiogenic (ANGPTL4 and SPHK1), inflammatory (NGF and IL1b) and pro-remodeling factors (MMP1 and MMP3) while collagen expression was reduced (Fig. 6).

### Synergistic regulation of inflammatory and degenerative factors in human tenocytes by cyclic stretching and mast cell presence

**Figure 7:**
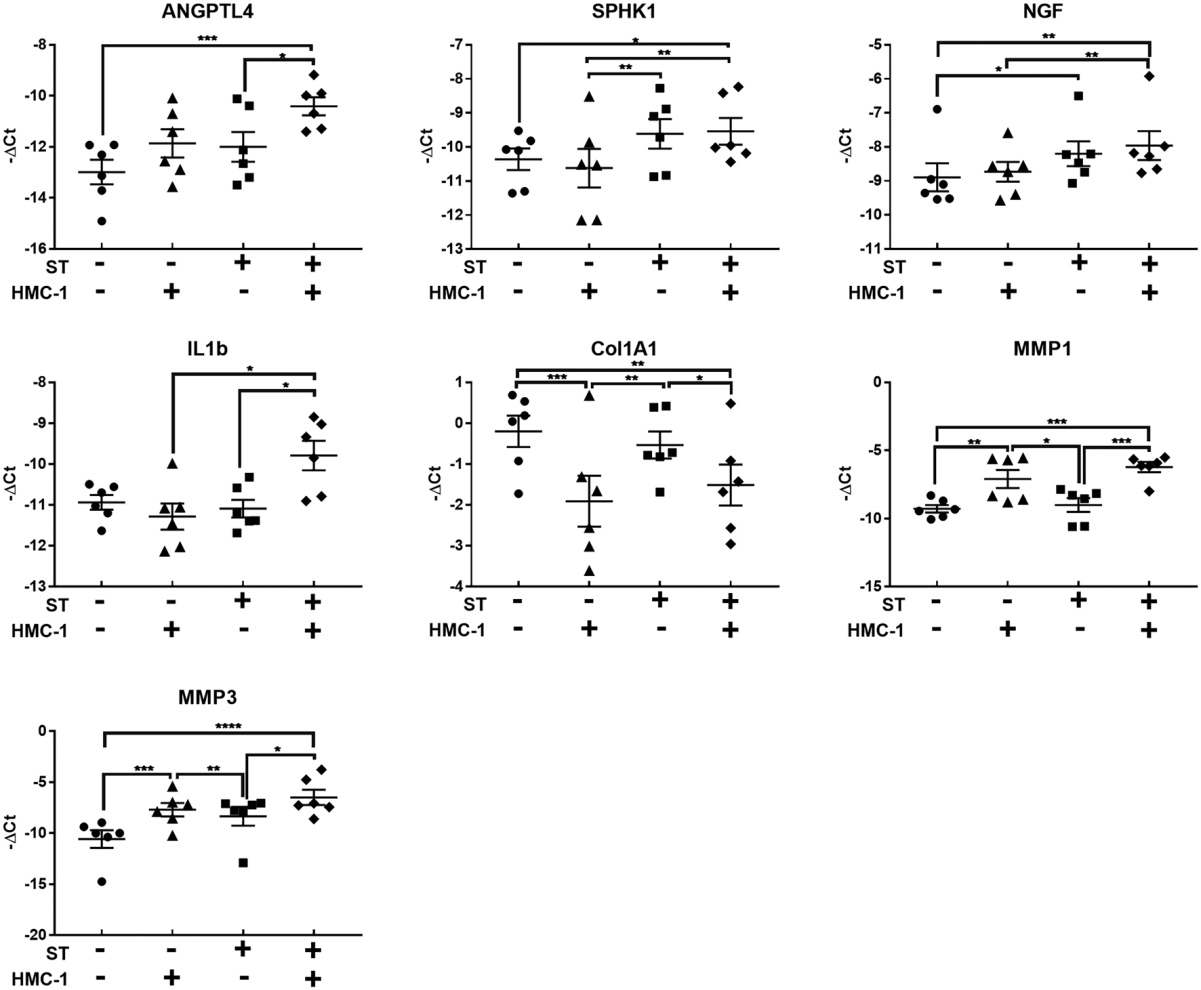
Synergistic impact of mechanical stretching and mast cells on tenocyte gene expression. The presence of HMC-1 in co-culture with human tendon cells leads to significant changes in the gene expression of tenocytes, particularly when subjected to mechanical stretching (ST) compared to non-stretched (NS) conditions. ANGPTL4: Angiopoietin-like 4; SPHK1: Sphingosine kinase 1; NGF: Nerve growth factor; Col1a1: Collagen type I αα1 chain; MMP1: Matrix metalloproteinase-1; MMP3: Matrix metalloproteinase-3; IL1b: Interleukin-1 β. One-way ANOVA followed by a post hoc Tukey's test; mean ± SE; *P ≤ 0.05; **P ≤ 0.01; ***P ≤ 0.001; ****P ≤ 0.0001; n = 6.

Further exploration of the crosstalk between human tenocytes and MCs in a mechanically active environment was conducted in a co-culture system. In this system, tenocytes, subjected to cyclic stretching, were simultaneously exposed to HMC-1 cells. Gene expression analysis revealed a synergistic impact resembling the observations made with mast cell degranulation triggered by MRGPRX2 agonists. Specifically, the co-culture induced a significant upregulation in the expression of inflammatory factors and proteolytic enzymes. Notably, collagen expression consistently decreased in the presence of HMC-1 (Fig. 7).

## Discussion

This study provides insights into the potential mechanisms of tendinopathy. Our findings highlight that repetitively stretched human tendon cells produce MC activators, including SP, which preferentially acts through the MRGPRX2 receptor. Activation of this receptor triggers MCs to migrate toward tenocytes as well as to degranulate. In turn, the factors released by these activated MCs promote the expression of inflammation-repair and degenerative markers in tenocytes, mirroring key aspects of the pathological characteristics of overuse tendinopathy.

Prior studies have reported an increased presence of MCs in tendinopathic human patellar^2^ and rotator cuff tendons^30^, but the precise mechanisms driving this phenomenon remained unexplained. Tenocytes, as fibroblast-like resident cells in tendon tissue, exhibit mechanical responsiveness, detecting and responding to applied strains through various mechanisms, including integrins and ion channels^31^. Mechanical loading can modulate the expression and release of various factors, such as cytokines and growth factors, influencing the surrounding tissues and cells including MCs^32^. Acute tendon injuries and inflammation also activate tendon resident cells, leading to increased mast cell presence and degranulation in the healing tendons^33^.

MCs originate from CD34 + pluripotent progenitor cells located in the bone marrow, and their differentiation is initiated by the binding of stem cell factor (SCF) to the Kit receptor^34,35^. These progenitor cells possess the capability to circulate in the bloodstream and migrate into tissues^36^. Differentiated human MCs retain the ability to migrate and proliferate within tissues^37^. In pathological conditions such as inflammatory disorders, chronic infections, and allergic reactions, an increased number of MCs is observed in tissues^4^. The existing literature lacks information on whether the increased mast cell count in tendinopathic tissue results from migration, cell proliferation, or infiltration of progenitors from the bloodstream. However, our in vitro model suggests that the infiltration of MCs in repetitive tendinopathy may primarily occur through cell migration rather than proliferation. This distinction provides a valuable perspective on the cellular dynamics underlying the observed increase in mast cell numbers in tendinopathic conditions.

Tenocytes are capable of producing a number of mast cell chemoattractants such as prostaglandins, chemokines, TGF-β isoforms and stem cell factor (SCF). We previously showed that cyclic stretching of human tenocytes^19,38,39^ increases the expression of Cox- 2 and the activity of TGF-β^12,40^. In addition to autocrine effects of the factors on tenocytes, they can stimulate mast cell migration and degranulation such as SP^21^. We have previously shown that SP is produced by tenocytes,^14^ that levels of SP in tendon are higher in patients with tendinosis^41^, and that SP production by tenocytes increases in response to mechanical loading both in vivo^42^ and in vitro^14^. Our results showed upregulation of SP and fibronectin in stretched tenocytes. These factors are known to play crucial roles in mast cell adhesion, migration, and degranulation^20,21^. This observation highlights their potential involvement in the interplay between tenocytes and MCs under mechanical stress. MRGPRX2 (also known as the mouse homologue MRGPRB2), a MC specific receptor, is only present in connective tissue MCs and notably absent in mucosal MCs^43^. This receptor plays a pivotal role in mediating IgE-independent MC degranulation triggered by SP, promoting neurogenic inflammation and pain^16^. Notably, this effect driven by SP occurs independently of the canonical SP receptor, NK1R. Inhibition or deletion of NK1R does not hinder the inflammatory response to SP, highlighting the unique role of MRGPRB2 in this process^16^. In our study, we demonstrated that inhibiting the NK1R receptor on HCM-1 cells did not alter their response to factors released from stretched tenocytes. Conversely, antagonizing MRGPRX2 suppressed the increased degranulation and migration of HMC-1 cells in response to stretched tenocytes. These findings highlight the distinctive contribution of MRGPRX2 in modulating mast cell responses, particularly in the context of mechanical stimuli from stretched tenocytes.

Fibronectin plays a crucial role in regulating mast cell adhesion to tenocytes through integrin receptors, ultimately leading to mast cell degranulation and increased cytokine release^19^. We conducted an adhesion assay to compare MCs' adhesion to fibronectin and collagen. In this assay, we used a concentration of fibronectin that was 10 times lower than that of collagen. Despite this, our findings, aligning with existing research^44^, demonstrate higher binding affinity of MCs for fibronectin compared to collagen, resulting in increased mast cell degranulation. While prior studies indicated integrin activation by mechanical stimuli as a trigger for mast cell degranulation^45^, inhibiting these receptors with RGDS did not change the HMC-1 response to factors released from stretched human tenocytes. Overall, these observations emphasize the potential role of SP binding to MRGPRX2, rather than fibronectin, in orchestrating the observed MCs responses to released factors from stretched human tenocytes.

Activation of MCs induce changes in pro-inflammatory factors and matrix remodelling phenotype in tendon, thereby potentially contributing to tendinopathy^5^. We previously showed MCs have a profound influence on fundamental tenocyte behaviours like collagen remodeling activity^46^. Our current study reveals a cross-talk between MCs and tenocytes in a repetitive overuse mechanical environment. Activation of MRGPRX2 on MCs promotes a degenerative phenotype characterized by decreased collagen expression and increased expression of proteolytic enzymes, MMPs. The synergistic effects of MCs and repetitive mechanical loading exacerbate the increased expression of neuroinflammatory and angiogenic factors in tenocytes, including ANGPTL4, SPHK1, NGF, and IL-1β. These factors have been implicated in promoting pathological features of overuse tendon injuries, such as aberrant neovascularization, and chronic pain, inflammation and tissue degeneration^40,47–51^.

Overall, our findings underscore the intricate interplay between mechanical stimuli and MC influence on tenocyte behavior. The synergistic effects of MCs and repetitive mechanical stimuli promote inflammatory and degenerative pathways in tendon cells through the activation of the SP receptor MRGPRX2. This suggests MC inhibitors may provide potential effective treatments for tendinopathy, as explored previously^52^. While our study identifies this mechanism for the first time, it's important to note the limitations of an in vitro model, which may not fully capture the complexity of tenocyte behavior in the tendon tissue matrix with the presence of other factors and cell types. Although our previous studies have shown a correlation between changes in mRNA levels and protein levels or activity of certain proteins in human tenocytes^12,13,53^, this study is limited to gene expression analysis at the mRNA level. This limitation arises from the restricted number of passages (3–6 passages) for human tenocytes, which prevented us from conducting additional experiments. Additionally, this model utilized a cultured mast cell line rather than primary mast cells from connective tissues due to challenges in maintaining and expanding normal mast cells. While these cell lines express key proteins found in normal mast cells, such as tryptase, chymase, high-affinity IgE receptor FcεRI, and MRGPRX2, their expression levels are significantly lower compared to the normal mast cells derived from human tissue or progenitor cells^22,54^. Moreover, mast cells within the tendon tissue matrix may directly sense mechanical stimuli during movement, which could trigger cell activation. These disparities may lead to different outcomes compared to those observed in our in vitro model. Further in vivo studies are essential to fully comprehend the role of MCs and associated mechanisms in overuse tendon injuries. These investigations will bridge the gap between in vitro findings and the dynamic tendon tissue environment, guiding the development of targeted treatments.

## Material and methods

### Cell culture

Human tendon cells were isolated from human hamstring tendon tissue, following previously established protocols^12^. Healthy hamstring tendons (semitendinosis) were donated by three males (age 34 ± 12) and three females (age 33 ± 9). The protocol received approval from the University of British Columbia Clinical Research Ethics Board (Certificate Number H10-00,220), and written informed consent was obtained from all donors. All methods in this study were performed in accordance with the relevant guidelines and regulations. Tenocytes were dissociated from the tissue matrix through a combination of mechanical and enzymatic digestion. Isolated cells were cultured in high-glucose Dulbecco’s modified Eagle’s medium (DMEM), supplemented with 10% fetal bovine serum, 2 mM L-glutamine, 100 units/ml penicillin, and 100 µg/ml streptomycin, in a humidified incubator with 5% CO2 at 37 °C. The isolated cells were cultured on adherent plastic tissue culture plates which exhibit a spindle-shaped phenotype (see Supplementary Fig. S1). This method of culture preferentially obtains and selects for internally located tendon fibroblasts^55^. Only cells from the third to sixth passage were used for experiments, because longer term culture of tenocytes leads to a gradual reduction in the expression of tendon-related genes^56^. In our previous study, we demonstrated that our method of human tenocyte culture isolates adherent cells expressing tendon cell markers, which reached peak expression between 3 to 6 cell culture passages^12^.

HMC-1 is a cell line isolated from a patient with mast cell leukemia. It harbors an activating mutation in KIT receptors, resulting in independent growth of these cells from kit ligand^57^. However, the major characteristics of mast cells, including expression of histamine and tryptase, remain intact^25,58^. HMC-1 cells were expanded in IMDM media supplemented with 10% serum, 1.2 mM α-thioglycerol, and 1X antibiotic–antimycotic. Subsequently, HMC-1 cells were resuspended in 2% serum high-glucose DMEM at a concentration of 10^6^ cells/ml. To induce activation of MRGPRX2 and subsequent MC degranulation, cells were exposed to 50 µg/ml compound 48/80 (C48/80)^59^ (Sigma, USA, # C2313) for 24 h. To inhibit C48/80-induced MRGPRX2 activation, 20 micromolar QWF^26^ (Tocris, USA, #6642) was added to the culture. NKR1, a conventional SP receptor, was inhibited using 25 μM L-733,060 (Tocris, USA, # 1145)^59^. To block integrins, 100 micromolar RGDS (Tocris Bioscience, USA, # 3498) was introduced into the HMC-1 culture.

### Cyclic stretching and co-culture system

To apply repetitive stretching to human tenocytes, we used the FlexCell system as previously described^12^. In this model, the expression of cytokines and growth factors in tenocytes responds dynamically to mechanical stimulation^12,14^. Isolated human tenocytes were cultured on collagen coated Bioflex plates (Flexcell International Corp., USA, #BF-3001C) with a density of 1.2 × 10^5^ cells per well. Following an overnight period of serum starvation, cells were subjected to equiaxial stretching using the FlexCell unit FX-6000 system (Flexcell International Corp., Hillsborough NC, USA), applying 10% strain at a frequency of 1 Hz equibiaxially for 6 h per day over a 3-day duration. This stretching protocol has been demonstrated to be well-tolerated by human tenocytes , showing no signs of cell death, and to promote the release of factors contributing to tendinopathic injuries^12^. Following the stretching protocol, both conditioned media and cells were harvested for subsequent analyses.

For co-culturing HMC-1 and human tenocytes during mechanical stretching, HMC-1 cells were resuspended in tenocyte medium (high glucose DMEM) supplemented with 2% serum at concentration of 2 × 10^5^ cells/ml. 1 ml of cell suspension was added into the tissue culture plate transwell inserts (VWR, US, #10769-186), which were then mounted on the Bioflex plate containing human tenocyte culture. The plates were then subjected to stretching for 6 h per day over 3 days, after which tenocytes were harvested for gene expression analysis.

### Transwell migration assay

To elucidate the impact of factors secreted by human tenocytes on mast cell migration, a transwell migration assay was used. HMC-1 cells were subjected to serum-free DMEM overnight to induce a state of starvation. Subsequently, these cells (at a concentration of 10^6^ cells/ml) were placed into the cell culture transwell insert with pore size of 8 µm (VWR, USA, #10769-242) while conditioned media from human tenocytes were introduced into the bottom compartment. Following an overnight incubation, the migrated cells were fixed in formaldehyde for 10 min, stained with crystal violet for 20 min, and observed under an upright microscope. Images were captured with 10x (ocular) ad 20x (objective) lenses, resulting in a total magnification of 200x, to document the migratory response.

Quantification of the migrated cells was performed to assess the influence of the secreted factors on mast cell migration. The number of migrated cells was determined, providing valuable insights into the migratory behavior of HMC-1 cells in response to the paracrine signals from human tenocytes. This transwell migration assay served as a robust methodology to investigate the intricate intercellular interactions and the regulatory role of tenocyte-secreted factors on mast cell migration.

### Adhesion assay

A 96-well microplate was coated with 50 µl of collagen (100 μg/mL) or fibronectin (10 μg/ml) solution per well overnight in an incubator. After blocking with 3% BSA for 1 h and washing twice with PBS, HMC-1 cells resuspended in DMEM with 2% serum at a concentration of 10^6^ cells/ml and added to the plate. Following a 6-h incubation, non-adherent cells were discarded, and microwells were gently washed with PBS. Cells were fixed with 4% formalin for 15 min, and stained with crystal violet for 20 min. Subsequently, the cells were washed with PBS, and the staining was released by lysing with a 30% acetic acid solution. The absorbance of the lysate was measured at 590 nm using a plate microreader.

### Cell proliferation assay

The proliferation of HMC-1 was measure by MTS assay. HMC-1 cells (10^6^ cells/ml) were incubated with conditioned media of human tenocytes in a 96 well flat bottom plate for 24 h. Then MTS solution was added to the well and incubated for 1–2 h. The absorbance was measure at 490 nm using a plate reader.

### Mast cell degranulation assay

The extent of HMC-1 degranulation was determined by quantifying the release of β-hexosaminidase. HMC-1 cells (10^6^ cells/ml) in DMEM were aliquoted in a 96-well plate and incubated with conditioned media from human tenocytes, QWF (20 μM), L-733,060 (25 μM), and RGDS (100 μM) overnight. Supernatant and cell lysates were separately incubated with p-nitrophenyl-N-acetyl-b-d-glucopyranoside (PNAG) (Sigma, USA, # 487052) for 1 h at 37 °C. After the addition of 0.1 M carbonate buffer, the absorbance was measured at 405 nm. The extent of degranulation was calculated as β-hexosaminidase % of total = [(abs Supernatant  −  abs blank)/(abs Supernatant − abs blank) + (abs lysateabs blank)] × 100.

### Gene expression analysis

mRNA expression was examined by RT-qPCR, following established procedures. Total RNA was purified using the Monarch Total RNA Miniprep Kit (New England Biolabs, USA, #T2010S), followed by cDNA transcription with a High-Capacity cDNA Reverse Transcription Kit (Applied Biosystems, USA, #4368814) according to manufacturer protocols. 10 ng cDNA was used for qPCR, run on the 7500 Fast Real-Time PCR System (Applied Biosystems, USA), using Luna Universal qPCR Master Mix (New England Biolabs, USA, #M3003). The primers used for RT-qPCR are listed in Table 1.  − ΔCt was calculated as – (Ct _Target_ – Ct _GAPDH_).

**Table 1:** List of primers used for real time qPCR.

Gene name	Abbreviation	Forward primer	Reverse primer
Angiopoietin-like 4	ANGPTL4	CTCCCGTTAGCCCCTGAGAG	AGGTGCTGCTTCTCCAGGTG
Sphingosine kinase 1	SPHK1	GCTGTGCCTTAGTGTCTACTT	TCCACCTTCTCACCCTTCT
Nerve growth factor	NGF	GTCATCATCCCATCCCATCTTC	CTGTGGCGGTGGTCTTATC
Collagen type I α1 chain	Col1a1	CACCAATCACCTGCGGTACAGAA	CAGATCACGTCATCGCACAAC
Matrix metalloproteinase-1	MMP1	CAGAAAGAGACAGGAGACATGAG	GAAGAGTTATCCCTTGCCTATCC
Matrix metalloproteinase-3	MMP3	GTGAGGACACCAGCATGA A	GACCACTGTCCTTTCTCCTAAC
Interleukin-1 β	IL1b	CAAAGGCGGCCAGGATATAA	CTAGGGATTGAGTCCACATTCAG
Glyceraldehyde 3-phosphate dehydrogenase	GAPDH	TCTTTTGCGTCGCCAGCCGAG	TGACCAGGCGCCCAATACGAC

### Statistical analysis

The statistical analysis of the data was conducted using paired Student t-test or one-way ANOVA, followed by Tukey’s post hoc comparison for multiple groups. To mitigate the impact of baseline response variations due to using different patients and experimental conditions, all data were analyzed pairwise. A significance threshold of *p* < 0.05 was applied, and values below this threshold were considered statistically significant. We determined the sample size needed for this study using G-power software (Version 3.1.9.6), with a power of 0.8 and a significance level of *p* < 0.05. Based on our prior data^12,46^ and other studies^22,44,60^, we calculated that a sample size ranging from 4 to 6 is required for gene expression, cell migration, MC degranulation, and adhesion analysis. Therefore, all experiments were conducted using 6 donors, consisting of 3 males and 3 females.

The data analysis was performed using GraphPad Prism software, and graphs were generated to illustrate the results. Micrographs and graphs were compiled and visually presented using Adobe Illustrator. All data points were depicted in the graphs, and specific details regarding the data analysis for each figure were incorporated into the respective figure captions to enhance clarity and facilitate a comprehensive understanding of the presented results. This meticulous approach ensures transparency in data representation and facilitates the interpretation of the findings in the context of each figure.

### Supplementary Information

Supplementary Information.

## Data Availability

The datasets used and/or analysed during the current study available from the corresponding author on reasonable request.
