# Supplementary material for: MRGPRX2-mediated mast cell activation by substance P from overloaded human tenocytes induces inflammatory and degenerative responses in tendons
Source: Sci Rep. 2024 Jun 12;14:13540. doi: 10.1038/s41598-024-64222-1 (PMC11169467; doi:10.1038/s41598-024-64222-1)
Supplement: Supplementary file 1 — Supplementary Information. [file 41598_2024_64222_MOESM1_ESM.docx]

**MRGPRX2-Mediated Mast Cell Activation by Substance P from Overloaded Human Tenocytes Induces Inflammatory and Degenerative Responses in Tendons**

Rouhollah Mousavizadeh‎^1^, Charlie M. Waugh‎^1^, Robert G. McCormack^2^, Brian E. Cairns^‎3^, Alex Scott‎^1*^

1. Department of Physical Therapy, Centre for Aging SMART, University of British Columbia, Vancouver, British Columbia, Canada
2. Department of Orthopaedics, University of British Columbia, Vancouver, British Columbia, Canada
3. Faculty of Pharmaceutical Sciences, University of British Columbia, Vancouver, British Columbia, Canada


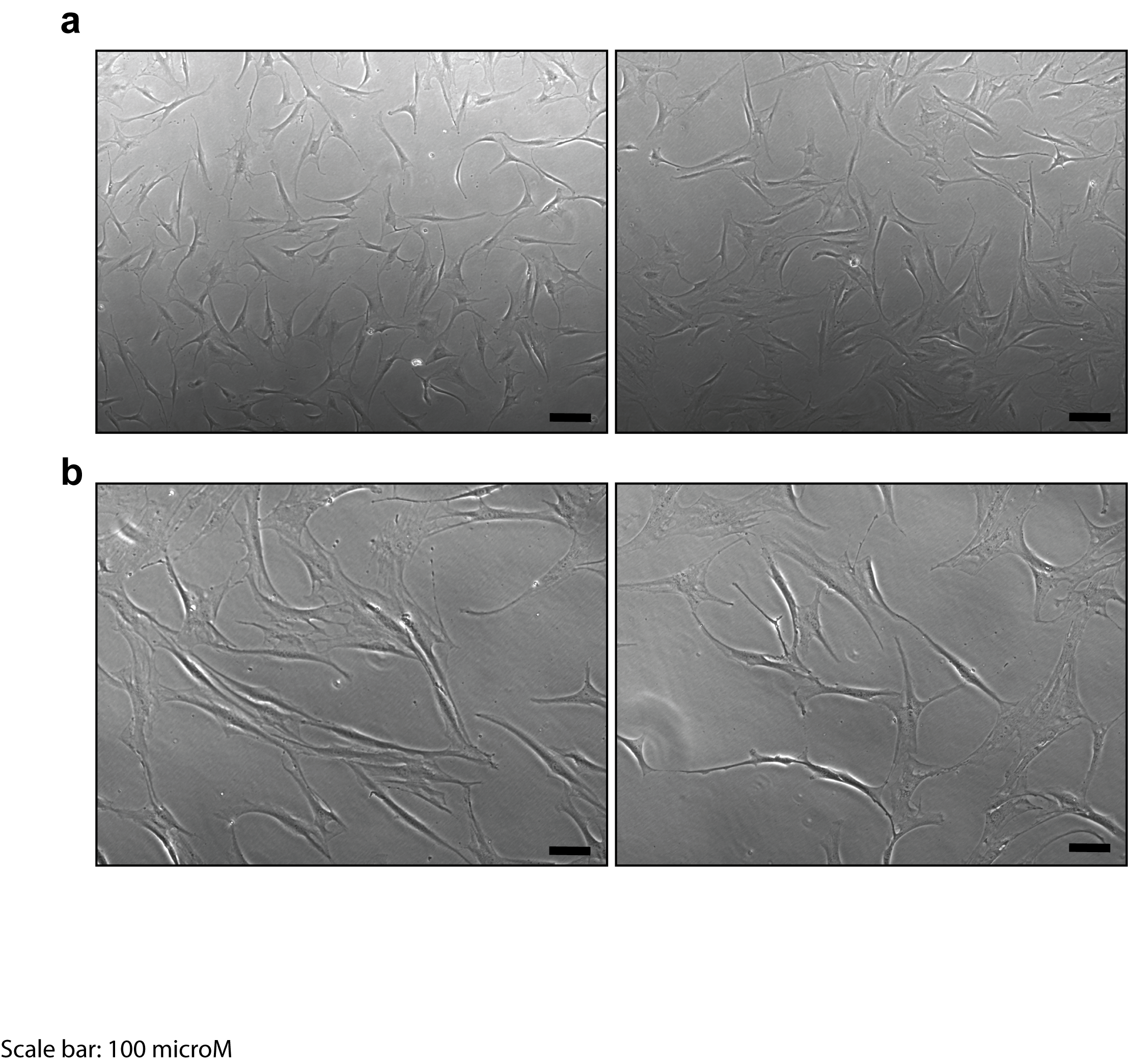


**Figure S1 Phase contrast images of human tenocyte culture.**

Adherent human tenocytes on plastic culture plates exhibit a spindle-shaped phenotype captured by phase-contrast microscopy at 50x (a) and 100x (b) magnifications.

Scale bars = 100 μm
